# Conservative surgical management of *in situ* subungual melanoma: long-term follow-up*

**DOI:** 10.1590/abd1806-4841.201645100

**Published:** 2016

**Authors:** Mariana Catalina De Anda-Juárez, María Abril Martínez-Velasco, Verónica Fonte-Ávalos, Sonia Toussaint-Caire, Judith Domínguez-Cherit

**Affiliations:** 1 Hospital General Dr. Manuel Gea González – Mexico City, Mexico.; 2 Instituto Nacional de Ciencias Médicas y Nutrición Salvador Zubirán – Mexico City, Mexico

**Keywords:** Follow-up studies, Melanoma, Nail diseases

## Abstract

Subungual melanoma represents 20% of all melanomas in Hispanic population. Here, we report the outcome of 15 patients with *in situ* subungual melanoma treated with resection of the nail unit with a 5-mm margin without amputation, followed up for 55.93 ± 43.08 months. The most common complications included inclusion cysts and nail spicules. We found no evidence of local or distant recurrences at the last visit of our follow up. Functional outcome was good, with only one patient reporting persistent mild pain. These results support functional, non-amputative surgical management of *in situ* subungual melanomas.

Subungual melanoma (SUM) is a subtype of acral melanoma. It represents 20% of all melanomas in Hispanic population and is the fourth most common nail tumor in our department.^1^ Prognosis and metastasis rates of SUM mainly depend on Breslow thickness as in other variants of melanoma.^2^ However, SUM is more often diagnosed in advanced stages.^3^

In early stages (radial growth), SUM presents as an irregular band of longitudinal melanonychia, sometimes with Hutchinson's sign (pigment extension around the nail apparatus). This clinical presentation generally corresponds to *in situ* or minimally invasive melanoma (less than 0.5 mm in Breslow depth).^3^ In more advanced stages, it is usually accompanied by nail dystrophy or can even become amelanotic, making the diagnosis more difficult.^4^ Dermoscopy reveals irregular deposition of pigment globules, irregular bands of melanonychia, and micro-Hutchinson sign.^5^ The gold standard for the diagnosis of SUM is nail matrix biopsy either by punch, tangential shaving, incisional nail matrix biopsy or lateral longitudinal biopsy.^6^ Recommended treatment consists of surgical excision and depends mainly on Breslow thickness.

The aim of the present study was to describe the clinical presentation, evolution, surgical technique, and outcome of a cohort of patients with *in situ* SUM treated with removal of the nail unit with 5 mm margins.

This study was conducted in the Dermatology Department of Hospital General Dr. Manuel Gea González in Mexico City. We performed a search in our histopathology database in order to retrieve all patients with diagnosis of *in situ* SUM from 1989 to 2013. All cases were confirmed by biopsy and treated with surgical excision of the nail unit with a 5 mm margin. Data obtained from records included: demographic data; time of evolution before final diagnostic biopsy; previous diagnosis and treatments; history of trauma; topography; clinical presentation; presence of Hutchinson's sign; nail dystrophy; dermoscopy (when available); and type of reconstruction.

Fifteen patients (9 females and 6 males; age 31.67 ± 20.9, range 4-66 years) were included and assessed with a physical exam and clinical photographs. Time of evolution until diagnostic biopsy was 79.4 ± 48.25 months (3 previously diagnosed as having naevi, 2 as racial melanonychia, and 1 treated as onychomycosis).

The most common clinical presentation was longitudinal melanonychia measuring 2-6 mm wide in 11 patients; with irregular distribution of pigment globules on dermoscopy. The 4 remaining presented with total melanonychia. Hutchinson's sign was present in 7 cases, and 5 patients had nail plate dystrophy, consisting of pachionychia and longitudinal riding. None of the patients reported history of trauma.

The right hand was involved in 5 patients (2 patients in the first finger, 2 patients in the fourth finger and one in the fifth finger), and the left hand in 4 patients (1 patient in the first finger and 3 in the fifth finger). The lower extremities were involved in the remaining 6 patients; 5 cases in the first finger of the right foot and one in the first finger of the left foot.

All patients were treated with complete removal of the nail apparatus with a 5 mm-margin and supra-periosteum depth resection. Fourteen patients were reconstructed with a full thickness skin graft, and one patient was treated with a banner flap (Figure 1).

**Figure 1:**
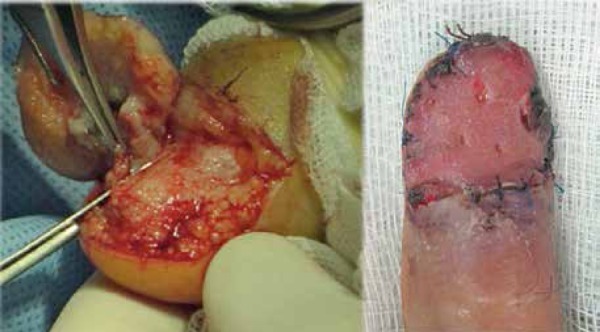
5-mm margin resection of the nail unit and reconstruction with full thickness skin graft

Follow up after surgical treatment was 55.93 ± 43.08 months (range 15 to 159 months). The most common complications were: nail spicules in 6 cases, inclusion cysts in 4 cases, persistent hypersensitivity of the digit in 4 patients, and persistent moderate pain when walking referred by one patient. Functional and cosmetic outcome was good in all of them with hyperpigmentation of the skin graft in 9 patients (Figure 2).

**Figure 2:**
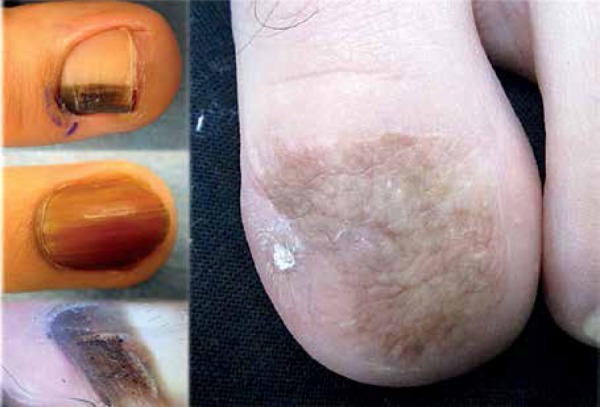
In-situ SUM: longitudinal melanonychia and total melanonychia. Dermoscopy shows irregular distribution of pigment globules. Hyperpigmentation of the graft and nail spicule on the lateral border

At the moment of this report, none of this patients presented local or distant tumor recurrence.

In the present series of patients, conservative management of *in situ* SUM with wide resection of the nail unit without amputation resulted in good functional and cosmetic outcomes in the long term, without any local or distant tumor recurrence. Complications of the surgical procedure were minor and only one patient had persistent pain. These results support wide resection treatment without amputation in patients with *in situ* SUM.

Recent studies by Lazar and Sureda support conservative or functional excision without amputation with wide margin resection of the nail unit in SUM and no recurrence after 2-4 years follow-up.^4,7^

Our findings support functional surgical treatment of patients with *in situ* SUM with a safe and good, result with only mild complications, without any recurrence after a minimal 15-month follow-up. This is a small series in Hispanic population that contributes by reporting results without amputation. Larger series and longer follow-up periods would contribute to confirm the safety of this procedure.
